# Study protocol of the RAND-study: a multicenter, prospective cohort study investigating response and adherence to nilotinib treatment in chronic myeloid leukemia

**DOI:** 10.1186/1471-2407-14-247

**Published:** 2014-04-08

**Authors:** Christel CLM Boons, Eleonora L Swart, Lonneke Timmers, Peter M van de Ven, Jeroen JWM Janssen, Jacqueline G Hugtenburg

**Affiliations:** 1 Department of Clinical Pharmacology and Pharmacy, VU University Medical Center, De Boelelaan 1117, 1081 HV Amsterdam, The Netherlands; 2 Department of Epidemiology and Biostatistics, VU University Medical Center, De Boelelaan 1118, 1081 HV Amsterdam, The Netherlands; 3 Department of Hematology, VU University Medical Center, De Boelelaan 1117, 1081 HV Amsterdam, The Netherlands; 4 The EMGO Institute for Health and Care Research, VU University Medical Center, Van der Boechorststraat 7, 1081 BT Amsterdam, The Netherlands

**Keywords:** Chronic myeloid leukemia, Nilotinib, Treatment outcome, Response, Medication adherence, Patients’ experiences

## Abstract

**Background:**

The antitumor drug nilotinib has a large inter- and intra individual variability in pharmacokinetics. Adherence to treatment may substantially influence plasma levels and has been recognized as the most important determinant of treatment failure in chronic myeloid leukemia (CML). A better understanding of the various factors contributing to the efficacy of treatment is essential for the development of interventions to optimize the treatment of chronic phase CML (CP-CML) with a protein kinase inhibitor like nilotinib.

**Methods/Design:**

In this multicenter prospective observational cohort study 70 adult patients with CP-CML starting treatment with nilotinib will be followed up for at least 12 months. Response to treatment is evaluated after 3, 6 and 12 months. Adherence is primarily assessed by counting the daily intake of nilotinib capsules by means of a medication event monitoring system (MEMS). Before the start of nilotinib treatment and after 3, 6 and 12 months, patients are asked to fill in a comprehensive questionnaire including topics on quality of life, side effects, attitude towards disease and medication, the patients’ appreciation of information received about the medication, and discontinuation, and trough plasma levels of nilotinib are measured.

**Discussion:**

The present study aims to get more insight into the efficacy of treatment with nilotinib and the various aspects that govern optimal response, of which adherence is a primary endpoint. We hypothesize that patients who experience inadequate response levels to nilotinib are less adherent. In addition, their plasma levels of nilotinib may be lower. We expect that our findings will be useful for health care professionals to support patients with the use of nilotinib in order to derive optimal benefit from their medication.

**Trial registration:**

Netherlands Trial Registry NTR3992.

## Background

Chronic myeloid leukemia (CML) is a malignant disease in which too many white blood cells belonging to the myeloid line of cells are produced in the bone marrow. CML is due to the growth and evolution of an abnormal clone of cells containing a chromosome rearrangement known as the Philadelphia chromosome (Ph+ cells). In the Ph chromosome a part of the *BCR* (‘breakpoint cluster region’) gene from chromosome 22 is fused with the *ABL* (‘Abelson leukemia virus’) gene on chromosome 9. The oncogenic *BCR-ABL* fusion gene encodes the BCR-ABL fusion protein. As a protein kinase it causes myeloid white blood cells to multiply uncontrollably [1].

Treatment of early or chronic phase Ph+ CML (CP-CML) with tyrosine kinase inhibitors (TKI) like imatinib results in very high response and survival rates [2]. Unfortunately, a considerable number of patients does not achieve adequate response levels and is at risk for disease progression. Causes of this lack of response include the presence of point mutations in the kinase domain of the BCR-ABL protein, increased expression of the *BCR-ABL* fusion gene and increased drug efflux mechanisms. Pharmacokinetic factors leading to suboptimal drug plasma concentrations may also affect the treatment result [3]. However, poor adherence to imatinib has now been recognized as the most important determinant of treatment failure [4-7]. The detrimental effect of poor adherence may be falsely interpreted as the development of resistance to TKI treatment. Reasons underlying poor adherence include the development of (serious) adverse effects, long duration of treatment and the absence of symptoms of the disease [8,9]. Resistance to imatinib as related to *BCR-ABL* point mutations has been largely overcome by using the 2^nd^ generation TKIs dasatinib and nilotinib [10,11]. Although data on long-term treatment results are not yet available, 2^nd^ generation TKIs, especially nilotinib, have been shown to be more effective than imatinib in first line therapy in attaining important endpoints as a complete cytogenetic response (CCyR), i.e. the absence of Ph^+^ cells in blood and bone marrow and a major molecular response (MMR), i.e. the near absence of *BCR-ABL* fusion mRNA transcripts (a reduction to ≤0.1% on the international scale) [12,13]. The latter endpoint is strongly predictive for long-term event-free survival [14]. First-line use of these TKI is therefore now recommended for intermediate and high risk patients [10,11]. On average, an MMR is attained at least one year earlier with the 2^nd^ generation TKIs as compared to imatinib [14,15].

A better understanding of the various factors contributing to the efficacy of treatment is essential for the development of interventions to optimize CP-CML treatment with a TKI. In the present study nilotinib treatment with the standard dose (300 mg twice daily) of patients with CP-CML is monitored as recommended by the current Dutch treatment guideline [10]. At present, treatment of CP-CML patients aims to achieve a lasting CCyR and MMR. However, on the basis of the more rapid onset of its effect than of imatinib [13], treatment with nilotinib could already result in a CCyR and MMR within 6 and 12 months after the start of treatment, respectively, instead of 12 and 18 months as stated in the guideline [10].

The present study aims to get more insight into the efficacy of treatment with nilotinib and the various aspects that govern optimal response, of which adherence is a primary endpoint. Adherence is primarily assessed by counting the daily intake of nilotinib capsules using a medication event monitoring system (MEMS). Patient experiences with drug use is assessed by means of a comprehensive questionnaire including topics on quality of life, side effects, attitude towards disease and medication, and the patients‘ appreciation of information received about the medication. Response to treatment is related to nilotinib trough plasma concentrations [16]. In contrast to venous puncture as used to obtain sufficient blood to evaluate efficacy, the Dried Blood Spot (DBS) sampling method requires only a very small quantity of blood that is obtained by means of a finger prick. Particularly at early stages of treatment this convenient and simple method is an ideal means to detect suboptimal nilotinib plasma concentrations that may occur as a result of non-adherence to treatment or drug-drug interactions.

### Objectives

The primary objective of the RAND-study (Response and Adherence to Nilotinib in Daily practice) is to assess whether CP-CML patients who respond to treatment with nilotinib (MMR within 12 months of treatment) differ from the non-responders in terms of adherence. Secondary objective is to evaluate whether responders and non-responders to nilotinib (molecular response after 3, 6 and 12 months of treatment) differ with respect to their plasma levels of nilotinib. Furthermore, the study aims to identify possible predictors for response to nilotinib treatment among hypothesized predictors that include occurrence of side effects, adherence, nilotinib blood levels, quality of life, attitude towards disease, beliefs and attitude towards medicines, patients‘ appreciation of information received about the medication, percentage of dose adjustment and discontinuation of treatment.

## Methods/Design

### Study design

The study is designed as a multicenter prospective observational cohort study. CP-CML patients starting treatment with nilotinib are followed up for at least 12 months as required to establish full cytogenetic responses in a relevant number of patients [10,14,15]. The study will end when the last patient has been followed for 12 months. In case of failure of treatment with nilotinib, treatment will be changed according to the guideline. Nilotinib treatment comprises the standard daily dose of nilotinib (300 mg twice daily) [3].

### Study population

The study population consists of 70 adult CP-CML patients starting treatment with a 2^nd^ generation TKI i.c. nilotinib.

### Study parameters

The primary research question is the comparison of patients responding to nilotinib treatment to the non-responders in terms of adherence. Response to nilotinib treatment is defined as presence of a MMR within 12 months after the start of first study medication. MMR is defined as ≤ 0.1% BCR-ABL/ABL (or any other validated house keeping gene) in the international scale, as measured by real-time quantitative PCR (RQ-PCR) [10]. The primary means of assessing adherence is the total intake of nilotinib capsules as counted by means of a MEMS taken as percentage of the number of pills prescribed over the 12 months follow-up period.

Secondary parameters include: rates of complete hematological response (CHR; the normalization of the blood cell count), CCyR (complete absence of Ph + cells in blood and bone marrow), and complete molecular response (CMR)(using RQ-PCR Ph + DNA cannot be detected); trough plasma level of nilotinib; potential drug-drug interactions; patient-reported side effects; adherence by means of telephonic pill count; adherence behaviour by means of the Medication Adherence Rating Scale (MARS); quality of life by means of the SF-12 Health Survey; attitude towards disease and medication by means of the Brief Illness Perception Questionnaire (IPQ) and the Beliefs about Medicines questionnaire (BMQ), resp.; patients‘ appreciation of information received about the medication by means of the Satisfaction with Information about Medicines Scale (SIMS); percentage of dose adjustment; patient-reported discontinuation; and patient demographics.

### Study procedures

#### Sampling of blood

After 3, 6 and 12 months of nilotinib treatment, patients are asked to provide blood samples by finger prick. Blood sampling will take place at the patient‘s home just before taking the next dose of nilotinib in order to obtain trough levels. Data on the exact time of administration of the previous dose are collected. Finger prick blood samples are collected using an automatic lancet device. Samples are collected from the fingertip. The first drop is discarded and the next 2 drops are collected to fill 2 8-mm premarked circles on the sampling paper (Whatman™, FTA™ DMPK-C (GE Healthcare), WB129243, obtained from VWR International BV, Amsterdam, The Netherlands). Volume of the blood drops of patients will be approximately 30 μl and blood spots of about 10-mm diameter are produced. The blood spots are allowed to dry at room temperature and packed in sealable plastic minibags. The DBS samples solely serve to monitor nilotinib blood levels. DBS samples are analyzed according to a recently developed method [17,18]. More detailed description of the analysis of DBS samples has been published elsewhere [17,18].

No other invasive intervention is performed for the present study. Response to treatment is regularly evaluated for treatment purposes in peripheral blood obtained by venapuncture and/or bone marrow after 3, 6 and 12 months of treatment. The results of the response to treatment are collected in the Case Report Form. Because Larson et al. [19] found that the occurrence of all-grade elevations in total bilirubin and to a lesser extend also of elevations in aspartate transaminase (ASAT) were higher in patients with higher nilotinib exposure, these parameters are determined as safety parameters at the same time as the regularly withdrawn venous blood samples for the measurement of response. The results of total bilirubin and ASAT are collected in the Case Report Form after 3, 6 and 12 months of treatment.

#### Pill count

Adherence is primarily assessed by counting the daily intake of nilotinib capsules by means of a MEMS, which is a microchip hidden in the lid of a medication box. Secondary assessment of adherence is by means of a telephonic pill count. Patients are contacted unannounced by the researcher by phone at the end of the 12 months follow-up period to count the number of remaining capsules at home. The count is performed using a standardized interview protocol. Pharmacy medication records and data on drug prescription in the medical file of the patient will be assessed. The total amount used by the patient is calculated by subtracting the amount in stock or returned to the pharmacy from the total amount collected from the pharmacy. The adherence rate is expressed as the percentage of drug taken by the patient of the total amount prescribed [20].

#### Review of the pharmacy medication records

The total number of delivered capsules, dose in milligram (mg) and dosing regimen of nilotinib is collected from the outpatient pharmacy of the hospital. Furthermore, pharmacy medication records of the past six months are collected for data on co-medication.

#### Review of the patient‘s medical file

Information on the disease, treatment plan and prescribed number of capsules, including dose, dose adjustments, (temporary) discontinuation or any other deviation from the prescribed treatment is retrieved from the patient‘s medical file.

#### Patient questionnaires

Before the start of nilotinib treatment and after 3, 6 and 12 months, patients are asked to fill in a comprehensive questionnaire on patients‘ experience with the use of nilotinib and their opinion on DBS. The questionnaire at baseline includes the factors date of birth, gender, living status, socio-economic status, non-prescription medication, quality of life, attitude towards disease and medication, and the side effects already perceived by the patient at baseline. The questionnaire at 3, 6 and 12 months includes non-prescription medication, quality of life, attitude towards disease and medication, side effects, adherence by means of the MARS, the patients‘ appreciation of information received about the medication, discontinuation and questions upon experiences with and opinions on the DBS. The following validated questionnaires will be used:

MARS: a validated self-report method to assess adherence behaviour. The MARS has five adherence statements, each scored on a 5-point Likert scale. This questionnaire assesses the frequency of non-adherent behaviour [21,22].

SF-12: quality of life is assessed with the SF-12 Health Survey. The SF-12 is a short version of the SF-36 and is a validated method to assess quality of life. The SF-12 is composed of 12 questions and standardized response choices, organized into eight scales: physical functioning, role limitations due to physical health problems, bodily pain, general health perceptions, vitality, social functioning, role limitations due to emotional problems, and general mental health. The standardized response choices vary dependent on the question from a five-item scale of always to never or a dichotomous scale from yes to no [23,24].

IPQ: a validated method to measure the attitude towards disease. The Brief IPQ consists of one open item and nine items on a continuous linear scale from 0 to 10 to assess perceptions. It assesses cognitive illness representations, emotional representations and illness comprehensibility [25].

BMQ: a validated questionnaire to assess beliefs and attitude towards medication. The BMQ consists of two five-item scale parts, the BMQ general and the BMQ specific. It assesses patients‘ beliefs and attitude towards the necessity of prescribed medication for controlling their disease and their concerns about potential adverse consequences of taking it. BMQ general measures the patients‘ beliefs and attitude towards medication in general. In the BMQ specific this is specified for the studied agent [26].

SIMS: the patients‘ appreciation of information given by physician/pharmacist/nurse practitioner about the medication is assessed with the SIMS questionnaire. The SIMS questionnaire is a validated method to assess the extent to which patients consider themselves (adequately) informed about their prescribed medication on a five-item scale [22].

### Ethical matters

The study is conducted according to the principles of the declaration of Helsinki (2008) and the Medical Research Involving Human Subjects Act (WMO), and has been approved by the Medical Research Ethics Committee (MREC) of the VU University Medical Center, Amsterdam, The Netherlands. In addition, the study has received approval for patient recruitment at the University Medical Center Utrecht, Medisch Spectrum Twente Enschede, University Medical Center Groningen, and Albert Schweitzer Hospital Dordrecht. Written informed consent will be obtained from all patients before enrolment.

### Statistics

#### Sample size calculation

In [6] a difference in nonadherence, measured as percentage of pills not taken of number prescribed, of 15.9% was found (mean nonadherence was 7.3% in responders and 23.2% in the non-responders) and the standard deviation of nonadherence was approximately 21.5%. Based on these numbers, the study described here is powered to detect a difference in mean adherence of three-quarters of the standard deviation. Under the assumption that adherence is normally distributed and 50 percent of the patients show a response, a total of 58 patients will yield 80% power to detect a difference of this size by means of an independent sample t-test and two-sided testing at a significance level of 5%. In case 60 percent of the patients show a response a total of 65 patients are needed. The sample size is set at 70 to take into account this uncertainty in number of patients responding and a small number of patients that become lost to follow-up.

#### Statistical analyses

Data will be analysed using SPSS 20.0 for Windows (SPSS Inc. Chicago, Illinois, USA). Baseline descriptive statistics will be presented as means (standard deviations) for normally distributed continuous data, medians and inter-quartile ranges for skewed continuous variables and as frequencies (percentage) for categorical variables. The primary objective is to compare adherence (percentage pills taken of those prescribed) between the responders and non-responders. In case, the normality assumption holds, this will be tested by an independent sample t-test. In case of skewed distribution for adherence, the non-parametric Mann–Whitney test will be used instead. Plasma level of nilotinib will be compared between responders and non-responders in the same manner. For the explorative assessment of predictors for response, we will use separate logistic regression models for each predictor. Logistic regression will also be used to identify possible confounders for adherence. Only the strongest predictors will be tested as confounding variables. Confounding variables are added as independent variables to the model relating response (dependent variable) to adherence (independent variable). Confounders are considered one-at-a-time as the number of patients falling in the smallest outcome category (of non-responders) is expected to include only between 20 and 30 patients, thereby adhering to the ten-event-per variable rule for logistic regression. For all analyses a two tailed significance level will be 0.05, all p-values below this level are considered statistically significant.

## Discussion

The present study is conducted to prospectively evaluate the relationship between the efficacy of treatment with nilotinib and adherence to treatment in CP-CML patients. We expect variability in adherence among the participating CP-CML patients. The evaluation of the influence of this adherence-variation on response may be complicated by inter-patient variability in pharmacokinetics of nilotinib. We hypothesize that patients who show a reduced molecular response to nilotinib are less adherent. In addition, these patients may have lower plasma levels of nilotinib. To obtain more insight into factors related to the efficacy of treatment, the effects of determinants are studied in an explorative manner. Especially, side effects may influence adherence, persistence and dose adjustments, and may thereby result in a reduced response. Furthermore, factors like beliefs about disease and medication or satisfaction upon information may influence adherence. The examination of these factors and patients‘ experiences with the DBS-method will give a broad insight into the use of nilotinib from the patients‘ point of view.

To date, no study has prospectively examined the influence of non-adherence on the outcome of nilotinib in CML. We expect that this study will provide valuable knowledge which will be useful for health care professionals to support patients with the use of nilotinib in order to obtain optimal response to nilotinib in daily practice.

## Abbreviations

ASAT: Aspartate transaminase; BMQ: Beliefs about Medicines Questionnaire; IPQ: Brief Illness Perception Questionnaire; CRF: Case Report Form; CML: Chronic Myeloid Leukemia; CP-CML: Chronic Phase Chronic Myeloid Leukemia; CCyR: Complete Cytogenetic Response; CHR: Complete Hematological Response; CMR: Complete Molecular Response; DBS: Dried Blood Spot; MMR: Major Molecular Response; MREC: Medical Research Ethics Committee; WMO: Medical Research Involving Human Subjects Act; MARS: Medication Adherence Rating Scale; MEMS: Medication Event Monitoring System; Ph+: Philadelphia positive; RQ-PCR: Real-time Quantitative-Polymerase Chain Reaction assay; SIMS: Satisfaction with Information about Medicines Scale; TKI: Tyrosine kinase inhibitors.

## Competing interests

The authors declare that they have no competing interests.

## Authors’ contributions

CB, ES, LT, JJ and JH participated in the design of the study concept and contributed to the protocol. PV contributed to the statistical section. JH and CB obtained funding and drafted the manuscript. CB will implement the multicenter protocol and collect the data. All authors read and approved the final manuscript.

## Pre-publication history

The pre-publication history for this paper can be accessed here:

http://www.biomedcentral.com/1471-2407/14/247/prepub
